# Strain-Specific Effects of *Bifidobacterium longum* on Hypercholesterolemic Rats and Potential Mechanisms

**DOI:** 10.3390/ijms22031305

**Published:** 2021-01-28

**Authors:** Jinchi Jiang, Caie Wu, Chengcheng Zhang, Qingsong Zhang, Leilei Yu, Jianxin Zhao, Hao Zhang, Arjan Narbad, Wei Chen, Qixiao Zhai

**Affiliations:** 1 State Key Laboratory of Food Science and Technology, Jiangnan University, Wuxi 214122, China; jiangjinchi@126.com (J.J.); 7160112075@vip.jiangnan.edu.cn (C.Z.); 7180112094@stu.jiangnan.edu.cn (Q.Z.); edyulei@126.com (L.Y.); jxzhao@jiangnan.edu.cn (J.Z.); zhanghao@jiangnan.edu.cn (H.Z.); chenwei66@jiangnan.edu.cn (W.C.); 2 College of Light Industry and Food Engineering, Nanjing Forestry University, Nanjing 210037, China; wucaie@njfu.edu.cn; 3 School of Food Science and Technology, Jiangnan University, Wuxi 214122, China; 4 International Joint Research Laboratory for Pharmabiotics & Antibiotic Resistance, Jiangnan University, Wuxi 214122, China; arjan.narbad@ifr.ac.uk; 5 National Engineering Research Center for Functional Food, Jiangnan University, Wuxi 214122, China; 6 Wuxi Translational Medicine Research Center and Jiangsu Translational Medicine Research Institute Wuxi Branch, Wuxi 214122, China; 7 (Yangzhou) Institute of Food Biotechnology, Jiangnan University, Yangzhou 225004, China; 8 Gut Health and Food Safety Programme, Quadram Institute Bioscience, Norwich Research Park Colney, Norwich, Norfolk NR4 7UA, UK; 9 Beijing Innovation Center of Food Nutrition and Human Health, Beijing Technology and Business University (BTBU), Beijing 100048, China

**Keywords:** *B. longum* strains, hypercholesterolemia, strain-specific, bile salt deconjugation, cholesterol assimilation, gut microbiota

## Abstract

Hypercholesterolemia is an independent risk factor of cardiovascular disease, which is among the major causes of death worldwide. The aim of this study was to explore whether Bifidobacterium longum strains exerted intra-species differences in cholesterol-lowering effects in hypercholesterolemic rats and to investigate the potential mechanisms. SD rats underwent gavage with each *B. longum* strain (CCFM 1077, I3, J3 and B3) daily for 28 days. *B. longum* CCFM 1077 exerted the most potent cholesterol-lowering effect, followed by *B. longum* I3 and B3, whereas *B. longum* B3 had no effect in alleviating hypercholesterolemia. Divergent alleviation of different *B. longum* strains on hypercholesterolemia can be attributed to the differences in bile salt deconjugation ability and cholesterol assimilation ability in vitro. By 16S rRNA metagenomics analysis, the relative abundance of beneficial genus increased in the *B. longum* CCFM 1077 treatment group. The expression of key genes involved in cholesterol metabolism were also altered after the *B. longum* CCFM 1077 treatment. In conclusion, *B. longum* exhibits strain-specific effects in the alleviation of hypercholesterolemia, mainly due to differences in bacterial characteristics, bile salt deconjugation ability, cholesterol assimilation ability, expressions of key genes involved in cholesterol metabolism and alterations of gut microbiota.

## 1. Introduction

Hypercholesterolemia is among the major causative factors for cardiovascular disease (CVD) [1]. CVD is regarded as a major health issue, accounting for 40% of all deaths in China in the past ten years; thus, hypercholesterolemia has become a serious challenge to the Chinese government for the prevention and control of these diseases [2,3]. In recent years, it has been widely reported that the reduction in total cholesterol and low-density lipoprotein cholesterol (LDL-C) in patients with hypercholesterolemia may reduce their CVD risk [4,5,6]. Previous studies have shown three-fold differences in the risk of CVD between people with hypercholesterolemia and those without [7]. Moreover, a 1% elevation in the serum cholesterol concentration has been found to cause a 2% to 3% increase in the incidence of CVD [8]. Among the main causes for hypercholesterolemia-related CVD is unhealthy changes in eating habits, such as increased intake of cholesterol and saturated fat, which disrupts the blood cholesterol metabolism and alters the composition and abundance of gut microbiota [9].

Currently, both pharmacological and non-pharmacological approaches, including drug treatments, dietary interventions and exercise, are clinically prescribed to control the serum cholesterol level [10]. However, due to the side effects of lipid-reducing drugs, contraindications for such medications, or personal preferences, many people prefer other functional foods to combat hypercholesterolemia. Therefore, it is crucial to find a safe and effective approach to alleviate hypercholesterolemia. The gut microbiota is reported to be related to metabolic diseases such as obesity, diabetes mellitus and hypercholesterolemia. Several studies have indicated that the composition of gut microbiota in hypercholesterolemic patients differs from that in healthy people, which is mainly characterized by the decreased relative levels of beneficial microbes, such as *Bifidobacterium* and *Lactobacillus*, and increased relative levels of pathogenic bacteria [11,12,13]. Therefore, probiotics are a new option for treatment of cholesterolemia.

Several animal and clinical trial studies have indicated the effectiveness of probiotics to treat hypercholesterolemia, either with single strain (*L. plantarum* ECGC 13110402, *L. reuteri* NCIMB 30242, or *B. longum* BB536) [14,15,16] or multiple strains (*L.plantarum* (CECT 7527,7528,7529), *L. plantarum* (ECGC 13, 110, 402), *Bifidobacterium* (*B. animalis subsp. lactis* MB 2409, *B. bifidum* MB 109 and *B. longum subsp. longum* BL04)) [14,17,18]. However, some strains (*L. rhamnosus* LC705, *Pediococcus pentosaceus* LP28) have been reported to be ineffective in alleviating hypercholesterolemia [19,20]. *B. longum*, as the most prevalent Bifidobacterium species in the human gastrointestinal tract, has been proven to be effective in protecting against CVDs, such as hypertension [21] and brain–gut-related diseases, such as chronic colitis and irritable bowel syndrome [22]. The underlying mechanism of the cholesterol-lowering effect of probiotics has been reported to be related to the expression of several genes involved in cholesterol metabolism [23,24,25]. FXR, CYP7A1 and SHP play important roles in the synthesis of bile acids from cholesterol, and LXR plays a role in reverse cholesterol transport [26,27]. Many hypotheses suggest that FXR is influenced by changes in the bile acid pool and that a decreased FXR expression downregulates SHP expression and upregulates CYP7A1 expression, both of which are bile acid synthesis rate-limiting enzymes [28]. Based on this background, we hypothesized that *B. longum* could prevent and treat hypercholesterolemia and that inter-strain differences exist in the treatment of hypercholesterolemia induced by a cholesterol-enriched diet in SD rats.

Therefore, we sought to explore whether *B. longum* presents strain-specific differences in the alleviation of hypercholesterolemia induced by a cholesterol-enriched diet in rats. Furthermore, the potential mechanisms of the inter-strain differences were evaluated, such as the basic physiological properties, the capacities of bile salt deconjugation and cholesterol assimilation, the mediated effects of key genes involved in cholesterol metabolism and the alteration of gut microbiota.

## 2. Results

### 2.1. Subsection Growth Characteristics of B. longum Strains in Vitro

**Figure 1:**
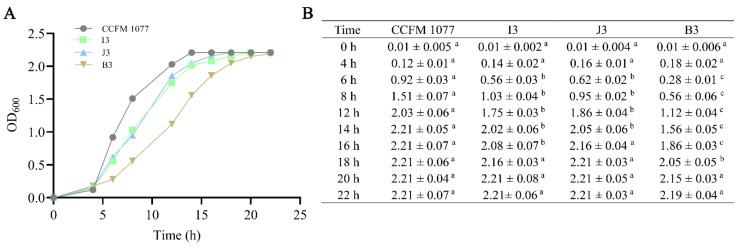
Growth status of four *B. longum* strains. (**A**) Growth curves of four *B. longum* strains. The X-axis is the culture time of four *B. longum* strains, and the Y-axis is the absorbance of culture liquid at 600 nm. The OD_600_ was tested three times at different time points (0, 4, 6, 8, 12, 14, 16, 18, 20 and 22 h). The data are expressed with the average of three OD_600_ values at each time point. (**B**) All OD_600_ values are means ± standard error of the mean. a, b, c: Means in the same row with different superscript letters are significantly different (*p* < 0.01) based on determination using a one-way ANOVA followed by Tukey multiple comparison test.

For determination of the growth characteristics of *B. longum* strains in vitro, the growth curve of the four strains was drawn. As is shown in Figure 1A, compared with other strains, *B. longum* CCFM 1077 is fastest in entering the logarithmic phase, followed by *B. longum* J3 and *B. longum* I3, whereas *B. longum* B3 is the slowest in entering the logarithmic phase. In addition, the statistical differences between different strains at different growth time are shown in Figure 1B.

### 2.2. Tolerance Ability of B. longum to Simulated Gastroenteric Fluid

The tolerance ability to gastric acid and bile salts of the four *B. longum* strains (CCFM 1077, I3, J3 and B3) was measured after cultivation at 37 °C in Whitley DG250 Anaerobic Workstation. As shown in Table 1, each of the four *B. longum* strains (*B. longum* CCFM 1077, *B. longum* I3, *B. longum* J3 and *B. longum* B3) showed high tolerance to the simulated intestinal environment of pH 2.5 and containing 0.3% bile acid (*p* < 0.01). 

### 2.3. Bile Salt Deconjugation and Cholesterol Assimilation Abilities of the B. longum Strains

Two properties of *B. longum* strains (bile salt deconjugation ability and cholesterol assimilation ability) were used to evaluate the cholesterol-lowering effect in vitro. As shown in Table 2, different properties were found in these four *B. longum* strains: *B. longum* CCFM 1077 shows both high abilities of bile salt deconjugation and cholesterol assimilation; *B. longum* I3 shows only high ability of bile salt deconjugation; *B. longum* J3 shows only high ability of cholesterol assimilation; *B. longum* B3 shows neither abilities of bile salt deconjugation or cholesterol assimilation.

(1) $$\mathrm{Bile}\;\mathrm{salt}\;\mathrm{deconjugation}\;\mathrm{ability}\;(\%)\;=\;\frac{C0-C1}{C0}\times 100$$

(2) $$\mathrm{Cholesterol}\;\mathrm{assimilation}\;\mathrm{ability}\;(\%)\;=\;\frac{100-\mathrm{residual}\;\mathrm{cholesterol}\;\mathrm{at}\;\mathrm{each}\;\mathrm{incubation}\;\mathrm{inverval}}{100}\times 100$$

### 2.4. The Effects of B. longum Strains on the Serum Lipids

**Figure 2:**
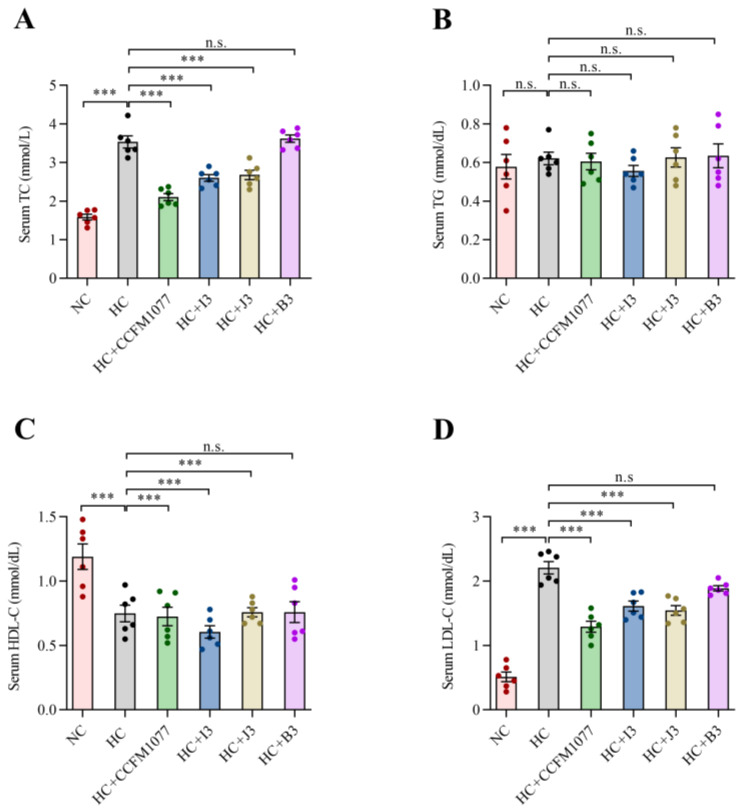
The effects of four *B. longum* strains on serum lipid levels of (**A**) TC, (**B**) TG, (**C**) HDL-C and (**D**) LDL-C in rats with hypercholesterolemia. *** represents *p* < 0.001, n.s represent non-significant with *p* > 0.05.

In terms of body weight, no significant differences were found among the three *B. longum* groups, HC group and NC group (Supplementary Figure S1). The serum lipid levels of the six groups are shown in Figure 2. Compared with the NC group, the HC group showed significantly higher levels in the serum TC and LDL-C levels. A significant decrease in the serum lipid levels was also observed in the CCFM 1077, I3 and J3 groups compared with the HC group (*p* < 0.001, Figure 1). Specifically, compared with the HC group, in the HC-CCFM 1077, HC-I3 and HC-J3 groups, the serum TC levels decreased by 44.44%, 30.56% and 27.77%; the serum LDL-C levels decreased by 40.91%, 31.82% and 27.27%, respectively. However, the serum HDL-C and TG levels showed no significant difference between the experimental groups. In addition, the serum lipid levels showed no difference between the HC-B3 and HC groups.

### 2.5. Effects of B. longum Strains on Fecal Bile Acid and Cholesterol Levels

**Figure 3:**
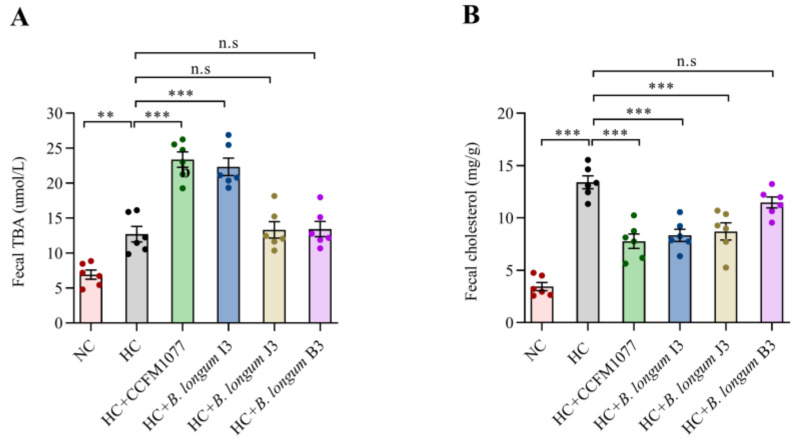
The effects of four *B. longum* strains on the levels of fecal bile acid (**A**) and fecal cholesterol (**B**). Data are expressed as means ± standard error of the mean in each group (*n* = 6). *** represents *p* < 0.001, ** represents *p* < 0.01, n.s represent non-significant with *p* > 0.05. *p*-values were determined using a one-way ANOVA followed by Tukey multiple comparison test.

The fecal bile acid and cholesterol levels in the different groups of rats on the last day are shown in Figure 3. The HC-CCFM 1077 and HC-I3 groups showed significantly higher levels of fecal total bile acid than the HC group (76.92% and 69.23%, *p* < 0.001, Figure 3A), and the HC-CCFM 1077 and HC-J3 groups showed significantly higher levels of fecal cholesterol (73.33% and 60.32%, *p* < 0.001, Figure 3B). However, *B. longum* B3 had no effect on the fecal excretion of bile acid and cholesterol compared with other strains.

### 2.6. Effects of B. longum Strains on Liver Gene Expression

**Figure 4:**
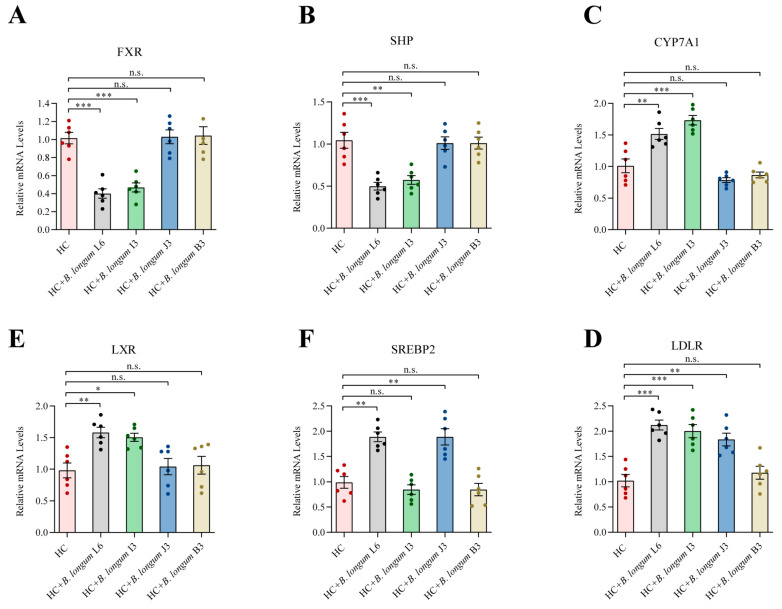
The effects of four *B. longum* strains on liver expression of genes involved in cholesterol and bile acid metabolism by RT-PCR. (**A**) FXR; (**B**) SHP; (**C**) CYP7A1; (**D**) LXR; (**E**) SREBP2; (**F**) LDLR. Each column represents the means ± standard error of the mean (*n* = 6). *** represents *p* < 0.001, ** represents *p* < 0.01, * represents *p* < 0.05, n.s represent non-significant with *p* > 0.05.

As shown in Figure 4, compared with the HC group, HC-CCFM 1077 and HC-I3 groups showed significantly downregulated *FXR* and *SHP* expression and upregulated *CYP7A1* and *LXR* expression (*p* < 0.001, Figure 4A–D). *SREBP2* expression was significantly upregulated by nearly two times in the HC-CCFM 1077 and HC-J3 groups (*p* < 0.01, Figure 4E); *LDLR* expression was also significantly upregulated in the HC-CCFM 1077, HC-I3 and HC-J3 groups (*p* < 0.001, Figure 4F).

### 2.7. Effects of the Four B. longum Strains on Gut Microbiota

**Figure 5:**
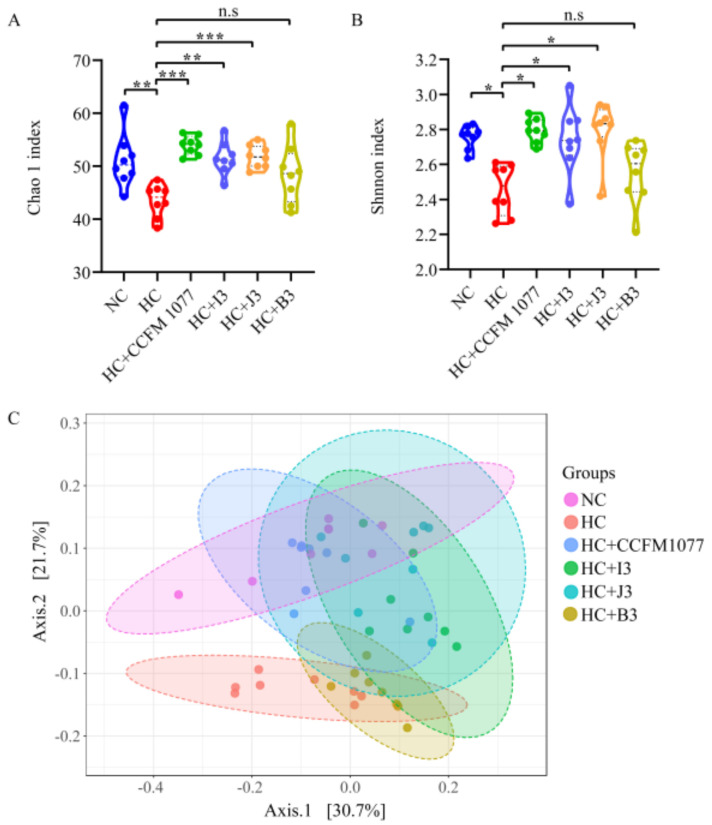
Effects of four *B. longum* strains (CCFM 1077, I3, J3, B3) on the overall structure of fecal microbiota. Alpha diversity was represented by (**A**) Chao 1 index and (**B**) Shannon index. Beta diversity was represented by (**C**) PCoA, based on weighted UniFrac distances. *** represents *p* < 0.001, ** represents *p* < 0.01, * represents *p* < 0.05, n.s represent non-significant with *p* > 0.05.

In our study, alpha diversity of gut microbiota was measured by Chao 1 and Shannon indexes. Both Chao 1 index and Shannon index of *B. longum* 1077, I3 and J3 treated rats were significantly higher than rats fed with a cholesterol-enriched diet, whereas *B. longum* B3 had no effect in these two indexes (Figure 5A,B). In terms of β-diversity, the gut microbiota between NC group and HC group were remarkably different; three *B. longum* groups (*B. longum* CCFM 1077, I3 and J3) improved the shift caused by the cholesterol-enriched diet, while *B. longum* B3 did not alter this shift (Figure 5C).

Through MiSeq sequencing analysis, we obtained 1,780,569 high-quality, classifiable 16S rRNA gene sequences in 36 fecal samples. The average sequence read was 12,864 bp per sample. Typical sequences were clustered, and a sequence similarity of 97% was considered as the cut-off. The number of OTUs per sample ranged from 966 to 8529.

**Figure 6:**
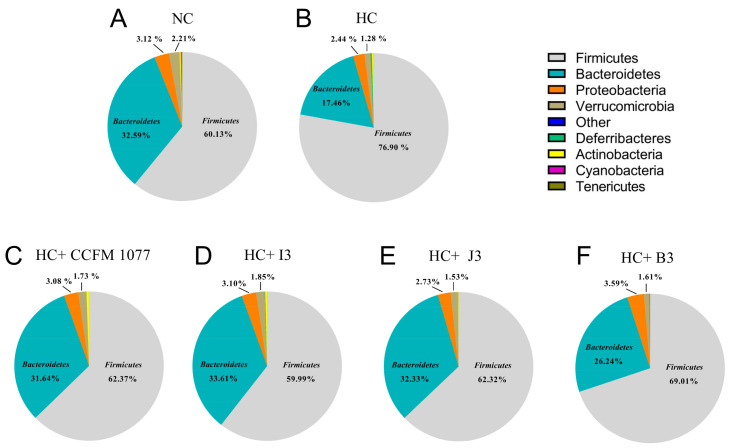
The relative abundance of the main phyla after consumption of high-cholesterol diet and administration of *B. longum* strains. (**A**) NC: non-cholesterol added diet; (**B**) HC: high-cholesterol diet; (**C**) HC+CCFM 1077: high-cholesterol diet + *B. longum* CCFM 1077; (**D**) HC+I3 high-cholesterol diet + *B. longum* I3; (**E**) HC+J3: high-cholesterol diet + *B. longum* J3; (**F**) HC+B3: high-cholesterol diet + *B. longum* B3.

In terms of the phylum level (Figure 6), the individual OTUs showed that the gut microbiota were mainly dominated by *Firmicutes*, *Bacteroidetes*, *Proteobacteria* and *Verrucomicrobia*, which accounted for 60.13%, 32.59%, 3.12% and 2.21% of the total gut microbiota, respectively. The composition of gut microbiota varied between different groups after feeding high-cholesterol diets to the groups for 28 days. Compared with the HC group, the HC-CCFM 1077, HC-I3 and HC-J3 groups showed a similar alteration, with a significant decrease in the relative abundance of Firmicutes (76.89% vs. 62.37%, 59.99% and 62.32%, respectively) and a significant increase in that of Bacteroidetes (17.46% vs. 31.64%, 33.60% and 32.33%, respectively) (*p* < 0.05). However, the HC+B3 group did not remarkably change the composition of gut microbiota at the phylum level.

**Figure 7:**
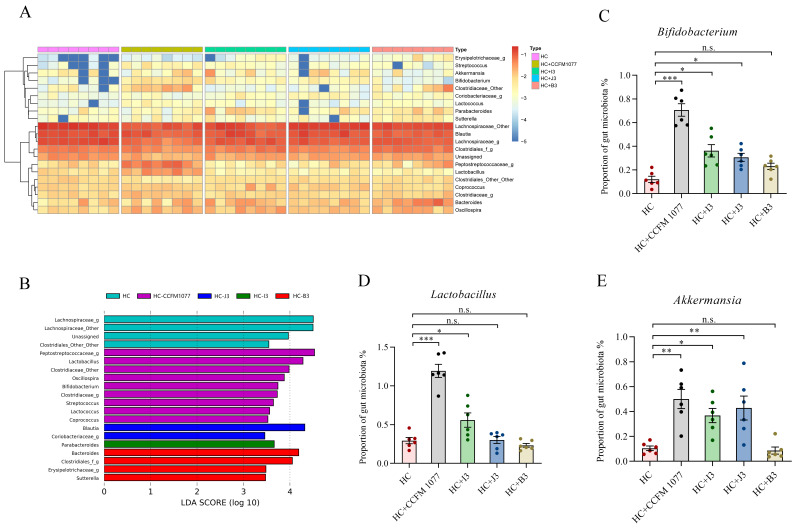
The relative abundance of the main genus after consumption of high-cholesterol diet and administration of *B. longum* strains. (**A**) Relative abundance (%) heatmap including all fecal samples. Sample clustering is shown on the left-hand side. (**B**) *LEfSe* analysis of the specific difference in the genus level in different groups. (**C**) The abundance of *Bacterium* in the feces. (**D**) The abundance of *Lactobacillus* in the feces. (**E**) The abundance of *Akkermansia* in the feces. Data are presented as means ± standard error of the mean, *n* = 6. *** represents *p* < 0.001, ** represents *p* < 0.01, * represents *p* < 0.05, n.s represent non-significant with *p* > 0.05.

At the genus level, genera with relative abundances over 0.1% are shown in Figure 7A. We categorized these genera into three main cluster groups (Cluster 1: low abundance; Cluster 2: high abundance; Cluster 3: medium abundance). Cluster 1 was dominated by Streptococcus and Bifidobacterium; Cluster 2 was dominated by *Lachnospiraceae* and *Blautia*; Cluster 3 was dominated by *Bacteroides* and *Lactobacillus*. In Cluster 1, compared with the HC group, *Streptococcus*, *Akkermansia*, *Bifidobacterium* and *Lactococcus* were more enriched in the HC-CCFM 1077 group. In Cluster 2, no different alterations were observed in these five groups. In Cluster 3, compared with the HC group, *Petostreptococcaceae* and *Lactobacillus* were observed to be increased in the HC-CCFM 1077 group. To further investigate the specific composition of gut microbita in different groups, the results of the *LEfSe* analysis are shown in Figure 7B. Compared with the HC group, the HC-CCFM 1077 group showed the greatest alterations in specific genera; among them, *Lactobacillus* and *Bifidobacterium* were the main contributors. *Blautia*, *Parabacteroides* and *Bacteroides* were also observed as the most specific in the HC-J3, HC-I3 and HC-B3 groups, respectively. In addition, we further analyzed the abundance of reported beneficial microbes (*Bifidobacterium, Lactobacillus* and *Akkermansia*) in the feces among these groups (Figure 7C–E). Compared with the HC group, these three genera in the HC-CCFM 1077 group and HC-I3 group were observed to be significantly increased (*p* < 0.001, *p* < 0.05); only *Bifidobacterium* and *Akkermansia* in the HC-B3 group were found to be significantly increased (*p* < 0.05), whereas these three genera in the HC-J3 group exerted no significant difference. Taken together, the cholesterol-lowering effect may be related to the alteration of the gut compositions and increase in the relative abundance of some genera, which is beneficial to human health.

### 2.8. Relationship between the Hypercholesterolemia-Alleviation Effects of B. longum Strains and Their Properties In Vitro

**Figure 8:**
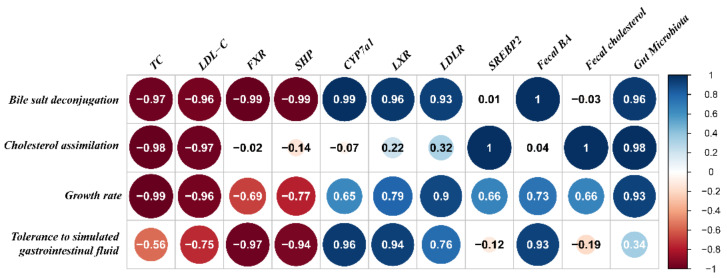
The correlation between the hypercholesterolemia-alleviation effects of *B. longum* strains and their properties in vitro.

In Figure 8, the Pearson correlation coefficients showed that the bile salt deconjugation ability, cholesterol assimilation ability and growth rate of strains were the most relevant indicators of the hypercholesterolemia-alleviation effects of *B. longum* strains. The serum lipid profiles (TC and LDL-C) and the expressions of some key genes involved in lipid metabolism (including FXR, SHP) were significantly negatively related to the bile salt deconjugation ability, with Pearson correlation coefficients below −0.95. In contrast, with the enhancement of bile salt deconjugation ability, the expressions of CYP7A1 and LXR, the fecal bile acid levels and the composition of gut microbiota exerted an upward trend. In terms of cholesterol assimilation ability, the TC and LDL-C levels showed a remarkable negative correlation (−0.98 and −0.97); however, the expression of *SREBP2*, the fecal cholesterol level and the composition of gut microbiota showed a significantly positive correlation (1, 1 and 0.98). Furthermore, the growth rate only had a positive correlation with the serum TC and LDL-C levels.

## 3. Discussion

Hypercholesterolemia is among the major risk factors for CVD, which is the leading cause of mortality worldwide [29,30]. To combat hypercholesterolemia, many companies and research institutions have developed lipid-lowering drugs, such as statins, fibrates, ezetimibe and berberine, via clinical research [31]. Despite the high therapeutic efficacies of these drugs, they all have adverse effects in humans, such as gastrointestinal symptoms and rashes [32]. Therefore, probiotics, which are microorganisms with many beneficial effects in humans, are being widely investigated for cholesterol-reducing functions in both animal experiments and clinic trials.

*B. longum* has been reported as the most prevalent *Bifidobacterium* species in the human gastrointestinal tract [33]. As a common resident of the gut, *B. longum* has been shown to possess a high intestinal colonization ability in human trials. The colonization ability of *B. longum* in the gut is related to the age of the people and the residential area [34]. For example, Fang and colleagues found that the abundance of *B. longum* in superlongevity people (aged over 90 years) from a longevity village (Bama, China) was higher than that in people from a normal area (Nanning, China) [35]. In addition, *B. longum* has been reported as the most universal species of the *Bifidobacterium* genus in the feces of Italian centenarians [36]. Several studies have investigated the cholesterol-lowering effect of *B. longum* strains, for example, *B. longum* BB536 was reported to significantly lower serum TC, LDL-C, VLDL and MDA [37]; however, *B. longum* BL 04 slightly altered the TC and LDL-C levels [18]. Although these *B. longum* strains were confirmed to alleviate hypercholesterolemia to some extent, the specific cholesterol-lowering effects were different. Therefore, the aim of our study was to explore whether *B. longum* presents inter-strain differences in the alleviation of hypercholesterolemia in vivo.

In our study, four *B. longum* strains (CCFM 1077, I3, J3 and B3) isolated from different superlongevity people (aged over 90 years old) were selected. Each of these four strains can survive adequately in the simulated intestinal conditions and can thus exert their effects in the human gut. Our results show that the *B. longum* strain exerted a strain-specific effect on cholesterol lowering. The precise cholesterol-lowering mechanism of probiotics is not fully understood, and many hypotheses have been proposed, such as bile salt deconjugation [38], cholesterol assimilation [39] and antioxidant activity of probiotics [40]. In our study, after intragastric administration of *B. longum* CCFM 1077, the content of both total bile acids and total cholesterol in the rat feces increased significantly, *B. longum* I3 only affected the fecal bile acid content and *B. longum* J3 only affected the fecal cholesterol content, whereas *B. longum* B3 had no effects on these two contents in feces. This finding indicates that the most significant alleviation effect of cholesterolemia by *B. longum* CCFM 1077 was related to the combination of high abilities of bile salt deconjugation and cholesterol assimilation in vitro.

Furthermore, to evaluate the underlying mechanism of the cholesterol-lowering effect of *B. longum*, we also studied the expression of several genes involved in cholesterol metabolism [23,24,25]. FXR, CYP7A1 and SHP play important roles in the synthesis of bile acids from cholesterol, and LXR plays a role in reverse cholesterol transport [26,27]. Many hypotheses suggest that FXR is influenced by changes in the bile acid pool and that a decreased FXR expression downregulates SHP expression and upregulates CYP7A1 expression, both of which are bile acid synthesis rate-limiting enzymes [28]. In this study, we found that compared with *B. longum* J3 and *B. longum* B3 administration, *B. longum* CCFM 1077 and *B. longum* I3 administration downregulated FXR and SHP expression and upregulated CYP7A1 and LXR expression (Figure 4A–D). This may be due to the high bile salt deconjugation ability of *B. longum* CCFM 1077 and *B. longum* I3, which hydrolyzed conjugated bile acids to free primary bile acids that are less efficiently reabsorbed in the intestine, thus increasing the fecal excretion of bile salts. This alteration in the bile acid pool downregulated FXR expression that in turn downregulated SHP expression and upregulated CYP7A1 expression, thus increasing cholesterol catabolism and bile synthesis. Consistent with this finding, GQ Wang et al. reported that FXR and SHP expression was decreased and CYP7A1 expression was increased in hypercholesterolemia mice after the oral administration of *L. plantarum* AR113 [41]. LDLR and SREBP2 are also important factors in the metabolism of cholesterol and other lipids [42]. In this study, LDLR expression in the *B. longum* CCFM 1077, *B. longum* I3 and *B. longum* J3 groups was upregulated compared with that in the *B. longum* B3 group, indicating that both bile salt deconjugation ability and cholesterol assimilation ability play an important role in reducing the serum cholesterol level by upregulating LDLR expression. However, we found that only *B. longum* CCFM 1077 and *B. longum* J3 increased SREBP2 expression, suggesting that cholesterol assimilation leads to cholesterol reduction via the upregulation of SREBP2 expression. This result is consistent with that of an earlier study that reported mediation of the cholesterol-lowering effect of soybean protein via the upregulation of LDLR expression [29,43].

Many studies have demonstrated that a high-cholesterol diet intervention disrupts the gut microbial balance [44,45]. In this study, we found that a high-cholesterol diet decreased the diversity and abundance of the gut microbiota in rats; in particular, the abundance of Firmicutes was higher and that of Bacteroidetes was lower in the HC group than in the *B. longum* groups. Therefore, we hypothesized that *B. longum* strains indirectly alleviate hypercholesterolemia by altering the gut microbiota. Previous studies have suggested that certain bacterial genera in the gut improve the health of the host by maintaining the gut microbial balance. *Bifidobacterium* and *Lactobacillus* were reported to lower the risk of CVD by reducing the liver cholesterol level [46], and *Akkermansia* could improve cholesterol levels in cases of metabolic syndrome [47]. Consistent with these studies, after intragastrically administering *B. longum* strains (CCFM 1077, I3 and J3) for 28 days, the abundance of *Bifidobacterium*, *Lactobacillus* and *Akkermansia* increased in the rat feces, thereby alleviating the adverse effects of high-cholesterol diet in vivo. However, this alteration was not observed in the *B. longum* B3 group. Taken together, our results show that only *B. longum* strains with either bile salt deconjugation ability or cholesterol assimilation ability can alleviate hypercholesterolemia by improving the composition of gut microbiota and increasing the abundance of health-promoting bacterial genera.

Physiological properties of strains in vitro are important for probiotic strains to take effect in human health. Correlation analysis showed a significant correlation of properties of strains in vitro (growth rate, bile salt deconjugation ability and cholesterol assimilation ability) with the cholesterol-alleviating effects. It has been reported that a strain with high bile salt deconjugation ability exerts the cholesterol-lowering effect by increasing the excretion of bile acid [48]. Chun-Feng et al. found that BSH-active *Lactobacillus casei* F0422 combined with Tween 80 with Cacl_2_ could increase the hypocholesterolemic effect in rats [49]. Guangqiang et al. found that *L. casei* pWQH01 (overexpression of bsh1) could significantly decreased the serum TC and LDL-C [41]. These results suggested that increased bile salt deconjugation may be among the factors of cholesterolemia mitigation. The high cholesterol assimilation ability of a strain means that more cholesterol is absorbed by the strain in the gut, and the assimilated cholesterol is excreted with large quantities of probiotic strain; for example, *B. longum* 5022 and *L. fermentum* LP4 with high cholesterol assimilation abilities have been reported to alleviate hypercholesterolemia [50,51]. The growth rate, as an indicator to evaluate how fast a strain grows, is also related to the hypercholesterolemia-alleviating effect. A fast growth rate contributes to the metabolism and proliferation of a strain and also increases the bile salt deconjugation ability and cholesterol assimilation ability.

In this study, remarkable differences in the bile salt deconjugation ability and cholesterol assimilation ability existed between the four *B. longum* strains. Among the *B. longum* strains, CCFM 1077 had both high abilities of bile salt deconjugation and cholesterol assimilation, I3 had only high bile salt deconjugation ability, J3 had only high cholesterol assimilation ability, whereas B3 had neither of these two abilities. Interestingly, in the animal experiments, *B. longum* CCFM 1077 exerted the most potent cholesterol-lowering effect, followed by *B. longum* I3 and B3, whereas *B. longum* B3 had no effect in alleviating hypercholesterolemia. This result supported that the strain-specific effects of *B. longum* strains on hypercholesterolemia rats mainly correlated with bile salt deconjugation and cholesterol assimilation abilities in vitro. In future, we will conduct clinical studies to further investigate the strain-specific effects of different probiotics on hypercholesterolemia.

## 4. Materials and Methods

### 4.1. The Culture Conditions and Growth Curve of Bacterial Strains

Four *B. longum* strains (CCFM 1077, I3, J3, B3) isolated from different superlongevity people (aged over 90 years old) were obtained from Culture Collections of Food Microbiology, Jiangnan University (Wuxi, China). To ensure the viable count of the probiotics before use, all four *B. longum* strains were reactivated three times by using 2% (*v*/*v*) inoculum in modified MRS (mMRS) broth supplemented with 0.5 ‰ (*w*/*v*) L-cysteine for 48 h in Whitley DG250 Anaerobic Workstation (37 °C). About 10^7^ CFU/mL of each *B. longum* strains was inoculated into fresh mMRS broth, then grown at 37 °C in anaerobic incubation workstation. OD_600_ values were measured every two hours. The horizontal axis in the diagram of growth curve shows the incubation time, and the vertical axis shows the corresponding absorbance values.

### 4.2. The Culture Conditions and Growth Curve of Bacterial Strains

The method used for testing the acid and bile tolerance of four *B. longum* strains has been previously described [50]. Briefly, *B. longum* was cultivated in mMRS broth at 37 °C for 18 h and then harvested by centrifugation (10,000× *g*, 4 °C, 10 min). After this, the cell pellets were suspended in phosphate-buffered saline solution, simulated bile salts (0.3% Oxigall dissolved in PBS, pH 8.0) or simulated gastric juice (3 mg of pepsin dissolved in 1 mL of 0.5% saline buffer, pH 2.5). The suspensions were incubated at 37 °C in simulated gastric juice for 1 h and then in simulated bile salts for 3 h. Finally, cells in the suspensions were harvested by centrifugation (10,000× *g*, 4 °C, 5 min) and resuspended in mMRS broth, followed by incubation at 37 °C for 48 h.

### 4.3. Quantitative Determination of Bile Salt Deconjugation Ability of B. longum Strains by HPLC

The bile salt deconjugation ability measurement of four *B. longum* strains was performed as previously described [52], with slight modifications. Briefly, cultures were grown in 10 mL of MRS broth (pH 6.5) by incubation at 37 °C for 18 h in the Whitley DG250 Anaerobic Workstation until a density of 1 × 10^9^ CFU/mL was reached. MRS broth (pH 6.5) containing 10 mM glycocholic acid (GCA; Sigma Chemical Co., St. Louis, MO, USA) was diluted 1:1 to a GCA concentration of 2.3 mg/mL, and 0.5 mL was added to the *B. longum* cultures. The cultures were then incubated for an additional 6 h under anaerobic conditions at 37 °C. The pH of the culture was then adjusted to 7.5 with KOH to stop the bile salt deconjugation ability. For HPLC quantification of GCA, the whole culture samples were first diluted 20 times in HPLC-grade methanol, 10 mL of which was injected into a C_18_ column (250 mm × 4.6 mm × 5 μm). In total, 30% 0.07-M sodium acetate (pH 3.0) and 70% methanol were used as the running buffer, and the flow rate was set at 1 mL/min. A Shimadzu SPD-10A UV-Vis detector was used to detect GCA at 205 nm. A standard stock solution of GCA was prepared by dissolving 10 mg GCA in 10 mL methanol. The concentration of stock solution was 100 μg/mL by diluting GCA in methanol. The standard was used as the reference control for GCA quantification. To plot the curve of bile salt deconjugation, the means of triplicate samples were calculated. Bile salt deconjugation ability was calculated as follows:

(3) $$\%\;\mathrm{Bile}\;\mathrm{acid}\;\mathrm{deconjugation}\;\;\left(C_{0}-C_{1}\right)/C_{0}\;\times \;100$$

where C_0_ is the GCA concentration of the control sample, and C_1_ is the GCA concentration of the *B. longum* sample.

### 4.4. Cholesterol Assimilation by B. longum Strains

The cholesterol concentration was determined by HPLC using the method described in a previous study [53], with slight modifications. After centrifuging the bacterial cultures at 7500g and 4 °C for 10 min, 1 μL of the culture supernatant was filtered and injected into the HPLC device (Agilent 1100, Agilent Technologies, Chandler, AZ, USA). The analysis was performed on a ZORBAX Eclipse XDB-C18 column (rapid resolution, 1.8 μm particle size, 4.6 × 50 mm, Agilent) with a variable wavelength UV/Vis detector. Acetonitrile at 2.5 mL/min was used for isocratic elution. Cholesterol was finally confirmed by the retention time of 4 min at 210 nm and was quantified by interpolation of the calibration curve. Standard solutions of various concentrations were prepared by diluting the cholesterol stock solution in MRS broth. Linearity was demonstrated from 0.01 to 0.1 g/L (r^2^ = 0.996). The limit of detection was 0.01 g/L.

(4) $$\%\;\mathrm{of}\;\mathrm{cholesterol}\;\mathrm{removed}\;\;\frac{100-residual\;cholesterol\;at\;each\;incubation\;inverval}{100}\times 100$$

### 4.5. Animals and Diets

Forty-eight SD rats (body weight, 220–240 g) were purchased from Shanghai SLAC Laboratory Animal Co., Ltd. (Shanghai, China). The rats were housed in stainless metal cages (two rats per cage) in a room maintained at 55% ± 5% relative humidity and 22 ± 2 °C under a 12 h light/dark cycle. After a 7 day adaptation to the new environment, the rats were randomly divided into the following six groups according to the diets: (1) non-added cholesterol diet (NC); (2) high-cholesterol diet (HC); (3) HC-diet plus *B. longum* CCFM 1077 (HC-CCFM 1077); (4) HC-diet plus *B. longum* I3 (HC-I3); (5) HC-diet plus *B. longum* J3 (HC-J3); and (6) HC-diet plus *B. longum* B3 (HC-B3). The experiment lasted for 28 days, during which the rats had free access to water and their group-specific diet. Each day, 2 mL of saline solution (0.85%) was administered to the NC and HC groups, whereas the HC-CCFM 1077, HC-I3, HC-J3 and HC-B3 groups received 2 mL (10^9^ cfu/mL) of their specific *B. longum* strain dissolved in saline solution (0.85%) by gavage. Dietary intake was monitored daily, and body weights were recorded at the end of the experiment (Figure S1). The components of diets are shown in Table 3. All experimental procedures complied with the Animal Care Committee of Jiangnan University (Identification Number: JN No. 20160823-20160929, Approved date: 25th July 2016), and were carried out under the guidelines set by the European Community (Directive 2010/63/EU).

### 4.6. Analysis of Serum Lipid Levels

After the 28 days of experiment, the rats were fasted overnight (12 h) and sacrificed under isoflurane. Serum was collected from their abdominal aorta using blood collection tubes (SLAC Laboratory Animal Co., Ltd., Shanghai, China) containing heparin as an anticoagulant. The tubes were then centrifuged at 1400 g at 4 °C for 15 min for serum separation, and the serum samples were stored at −80 °C until analysis. The serum total cholesterol (TC), triglyceride (TG), high-density lipoprotein cholesterol (HDL-C) and LDL-C levels were measured using a Biochemical Analyzer (Beckman Coulter, Brea, CA, USA) [54].

### 4.7. Analysis of Fecal Cholesterol and Bile Acid Contents

On the last day of the experiment, the feces of each rat were collected and immediately stored at −80 °C until further analysis. Fecal cholesterol and bile acid contents were measured using commercial kits (Sangon Biotech, Shanghai, China).

### 4.8. Extraction of Liver RNA and RT-PCR Analysis

The total RNA samples from rat livers were extracted according to the manufacturer’s protocol (TaKaRa Bio, Otsu, Japan). The mRNA levels of sterol regulatory element-binding protein 2 (SREBP2), cholesterol 7α-hydroxylase (CYP7A1), liver X receptor (LXR), farnesoid X receptor (FXR) and low-density lipoprotein receptor (LDLR) were measured as described previously [50]. Total RNAs were extracted with TRIzol (Thermo Scientific, Wilmington, DE, USA), and cDNAs were synthesized by reverse transcription (TaKaRa Bio, Otsu, Japan). The quantitative real-time polymerase chain reaction (RT-qPCR) used the SYBR Premix Ex TaqII (TaKaRa Bio, Otsu, Japan) and the appropriate primers. The primer sequences which were obtained from Sangon Biological Engineering (Shanghai, China) are shown in Table 4. The thermocycler conditions used were 95 °C for 30 s, followed by 45 cycles of 95 °C for 5 s and 60 °C for 30 s.

### 4.9. MiSeq Genome Sequencing Analysis of Community Structures

Fecal microbial DNA was extracted using a FastDNA SPIN Kit for Soil (MP Biomedical, catalog No.6560-200) in accordance with the manufacturer’s instructions. Subsequently, 16S rRNA sequencing was performed, and the reads were analyzed using the QIIME pipeline [55]. Reads shorter than 200 bp were removed after screening the raw sequences, and sequenced pair-end reads overlapping by longer than 10 bp and without any mismatch were assembled. The sequences with similarity of more than 97% were defined as operational taxonomical units (OTUs) for assembly of high-quality sequences using the QIIME software (http://qiime.sourceforge.net/).

### 4.10. Correlation Test

R language was used to evaluate the correlation between the significantly altered indicators in the hypercholesterolemia-alleviating effect and the properties of strains in vitro. The data were divided into two matrices: an in vitro matrix and in vivo matrix. The corrplot package was used to analyze the correlations between these two matrices. Pearson’s correlation coefficients were calculated to quantify the correlations between the indicators.

### 4.11. Statistical Analysis

All of the data are expressed as the mean ± standard error of the mean. Statistical significance was determined by one-way analysis of variance followed by a Tukey multiple comparison test using GraphPad Prism 8.0 (GraphPad Software Inc., San Diego, CA, USA) and SPSS version 16.0 software (SPSS, Inc., Chicago, IL, USA).

## 5. Conclusions

By measuring serum lipid profiles (TC, TG, LDL-C and HDL-C), it was found that *B. longum* CCFM 1077 exerted the most potent cholesterol-lowering effect, followed by *B. longum* I3 and B3, whereas *B. longum* B3 had no effect in alleviating hypercholesterolemia. Furthermore, *B. longum* CCFM 1077, I3 and B3 significantly altered the diversity and composition of gut microbiota. These results suggested that *B. longum* (CCFM 1077, I3, J3 and B3) exerted strain-specific effects in alleviation of hypercholesterolemia. The potential causes of the strain-specific effects in alleviation of hypercholesterolemia might be the differences in the bile salt deconjugation ability, cholesterol assimilation ability and growth rate of different strains, which had different effects on the intestinal microenvironment and expressions of key genes involved in the lipid metabolism.

## Figures and Tables

**Table 1:** Tolerance of four *B.longum* strains to simulated gastroenteric environments.

Strain	Initial Survival in PBS	Survival after 2 h at pH 2.5 in SGJ		Survival after 3 h at pH 8.0 in 0.3% Oxigall	
	Mean Counts (Log CFU/mL)	Mean Counts (Log CFU/mL)	Survival Rate	Mean Counts (Log CFU/mL)	Survival Rate
*B. longum* CCFM 1077	9.76 ± 0.06 ^a^	7.97 ± 0.18 ^b^	81.66%	6.36 ± 0.12^c^	65.16%
*B. longum* I3	9.23 ± 0.24 ^a^	7.56 ± 0.14 ^b^	81.91%	6.08 ± 0.08 ^c^	65.87%
*B. longum* J3	9.32 ± 0.18 ^a^	7.86 ± 0.26 ^b^	84.33%	5.95 ± 0.12 ^c^	63.84%
*B. longum* B3	9.58 ± 0.08 ^a^	6.98 ± 0.23 ^b^	72.86%	6.24 ± 0.18 ^c^	65.14%

All values are means ± standard error of the mean. a,b: Means in the same row with different superscript letters are significantly different (*p* < 0.01) based on determination using a one-way ANOVA followed by Tukey multiple comparison test. % survival = final (CFU/mL)/control (CFU/mL) × 100% survival indicates that the growth rate of the strain was not affected by the treatment. PBS: Phosphate Buffer solution. SGJ: Simulated gastric juice.

**Table 2:** Bile salt hydrolysis ability and cholesterol assimilation ability of *B. longum* strains.

Strain	Bile Salt Hydrolysis Ability (%)	Cholesterol Assimilation Ability (%)
*B. longum* CCFM 1077	98.66 ± 0.65 ^a^	97.68 ± 1.03 ^a^
*B. longum* I3	97.36 ± 0.36 ^a^	0.96 ± 0.16 ^b^
*B. longum* J3	1.01 ± 0.02 ^b^	99.36 ± 0.32 ^a^
*B. longum* B3	1.12 ± 0.06 ^b^	0.82 ± 0.15 ^b^

All values are means ± standard error of the mean. Means in the same column with different superscript letters are significantly different (*p* < 0.01) based on the analysis using a one-way ANOVA followed by a Tukey multiple comparison test. C0 is the GCA concentration of the control sample, and C1 is the GCA concentration of the *B. longum* sample.

**Table 3:** Composition of the experimental diets.

Ingredient	Cholesterol-Free Diet (g/kg)	Cholesterol-Enriched Diet (g/kg)
Cornstarch	465.692	459.442
Dextrinized cornstarch	155	155
Casein	140	140
Sucrose	100	100
Soybean oil	40	40
Cellulose	50	50
Choline biartrate	2.5	2.5
L-Systine	1.8	1.8
t-Butylhydroquinone	0.008	0.008
Mineral	35	35
Vitamin	10	10
Cholesterol	-	5
Sodium cholate	-	1.25

Cholesterol-free and cholesterol-enriched diets were both bought from Trophic Animal Feed High-Tech Co., Ltd., Nantong, China.

**Table 4:** Primer sequences used for RT-PCR in this study.

Gene	Forward Primers (5′-3′)	Reverse Primers (5′-3′)
FXR	CCAACCTGGGCTTCTACCC	CACACAGCTCATCCCCTTT
SHP	TCTGCAGGTCGTCCGACTATTC	AGGCAGTGGCTGTGAGATGC
CYP7A1	ATTCCATACCTGGGCTGTGC	ATGTTTTCAGTGGTATTTCC
LXR	CTCTTCTTGCCGCTTCAGTT	AGGAGTGTCGACTTCGCAAA
Srebp 2	AGCAGCAGGTGCAGACGGTA	CATCTGTCTTCAGCGTGGTC
LDLR	AGCAGTGAGTGTATCCATCG	AATGCAGGAGCCATCTGCAC
β-actin	GGCTGTATTCCCCTCCATCG	CCAGTTGGTAACAATGCCATGT

FXR = farnesoid X receptor; SHP= small heterodimer partener; CYP7A1 = cholesterol 7α-hydroxylase; LXR = liver X receptor; Srebp2 = sterol regulatory element-binding protein 2; LDLR = low-density lipoprotein receptor.

## Data Availability

No new data were created or analyzed in this study. Data sharing is not applicable to this article.

## Supplementary Materials

The following are available online at https://www.mdpi.com/1422-0067/22/3/1305/s1, Figure S1: Body weight on the final day.

## Abbreviations

*B. longum*	*Bifidobacterium longum*
*L. plantarum*	*Lactobacillus plantarum*
*B. bifidum*	*Bifidobacterium bifidum*
*L. rhamnosus*	*Lactobacillus rhamnosus*
BSH	Bile salt hydrolysis
FXR	farnesoid X receptor
SHP	small heterodimer partner
CYP7A1	cholesterol 7α-hydroxylase
LXR	liver X receptor
SREBP2	sterol regulatory element binding protein 2
LDLR	low density lipoprotein receptor
