# Supplementary figures and images for: Strain-Specific Effects of Bifidobacterium longum on Hypercholesterolemic Rats and Potential Mechanisms
Source: Int J Mol Sci. 2021 Jan 28;22(3):1305. doi: 10.3390/ijms22031305 (PMC7866116; doi:10.3390/ijms22031305)

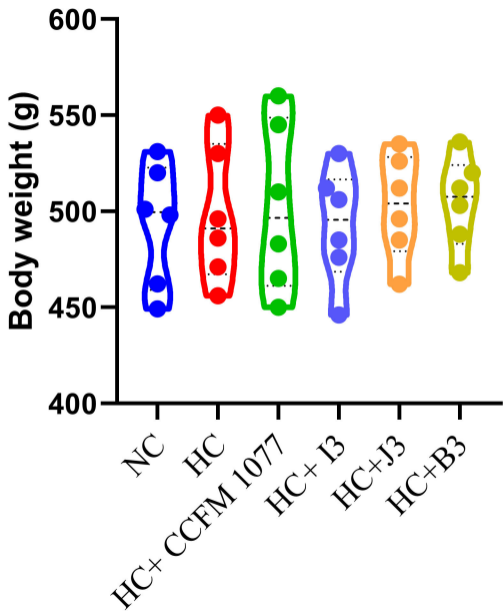

Supplement: Supplementary file 1 [file ijms-22-01305-s001.pdf]
